# Reduced Alcohol Consumption and Major Adverse Cardiovascular Events Among Individuals With Previously High Alcohol Consumption

**DOI:** 10.1001/jamanetworkopen.2024.4013

**Published:** 2024-03-28

**Authors:** Dong Oh Kang, Dae-In Lee, Seung-Young Roh, Jin Oh Na, Cheol Ung Choi, Jin Won Kim, Eung Ju Kim, Seung-Woon Rha, Chang Gyu Park, Ye-Seul Kim, Yonghwan Kim, Hyo-Sun You, Hee-Taik Kang, Eunseo Jo, Jinseob Kim, Jae-woo Lee, Jin-Man Jung

**Affiliations:** 1 Cardiovascular Center, Korea University Guro Hospital, Korea University College of Medicine, Seoul, Republic of Korea; 2 Department of Family Medicine, Chungbuk National University Hospital, Chungju, Republic of Korea; 3 Department of Family Medicine, Severance Hospital, Yonsei University College of Medicine, Seoul, Republic of Korea; 4 Department of Statistical Analysis, Zarathu Co Ltd, Seoul, Republic of Korea; 5 Department of Family Medicine, Chungbuk National University College of Medicine, Chungju, Republic of Korea; 6 Department of Neurology, Korea University Ansan Hospital, Korea University College of Medicine, Ansan, Republic of Korea

## Abstract

**Importance:**

Cardiovascular benefits of mild to moderate alcohol consumption need to be validated in the context of behavioral changes. The benefits of reduced alcohol consumption among people who drink heavily across different subtypes of cardiovascular disease (CVD) are unclear.

**Objective:**

To investigate the association between reduced alcohol consumption and risk of major adverse cardiovascular events (MACEs) in individuals who drink heavily across different CVD subtypes.

**Design, Setting, and Participants:**

This cohort study analyzed data from the Korean National Health Insurance Service–Health Screening database and self-reported questionnaires. The nationally representative cohort comprised Korean citizens aged 40 to 79 years who had national health insurance coverage on December 31, 2002, and were included in the 2002 to 2003 National Health Screening Program. People who drank heavily who underwent serial health examinations over 2 consecutive periods (first period: 2005-2008; second period: 2009-2012) were included and analyzed between February and May 2023. Heavy drinking was defined as more than 4 drinks (56 g) per day or more than 14 drinks (196 g) per week for males and more than 3 drinks (42 g) per day or more than 7 drinks (98 g) per week for females.

**Exposures:**

Habitual change in heavy alcohol consumption during the second health examination period. People who drank heavily at baseline were categorized into 2 groups according to changes in alcohol consumption during the second health examination period as sustained heavy drinking or reduced drinking.

**Main Outcomes and Measures:**

The primary outcome was the occurrence of MACEs, a composite of nonfatal myocardial infarction or angina undergoing revascularization, any stroke accompanied by hospitalization, and all-cause death.

**Results:**

Of the 21 011 participants with heavy alcohol consumption at baseline (18 963 males [90.3%]; mean [SD] age, 56.08 [6.16] years) included in the study, 14 220 (67.7%) sustained heavy drinking, whereas 6791 (32.2%) shifted to mild to moderate drinking. During the follow-up of 162 378 person-years, the sustained heavy drinking group experienced a significantly higher incidence of MACEs than the reduced drinking group (817 vs 675 per 100 000 person-years; log-rank *P* = .003). Reduced alcohol consumption was associated with a 23% lower risk of MACEs compared with sustained heavy drinking (propensity score matching hazard ratio [PSM HR], 0.77; 95% CI, 0.67-0.88). These benefits were mostly accounted for by a significant reduction in the incidence of angina (PSM HR, 0.70; 95% CI, 0.51-0.97) and ischemic stroke (PSM HR, 0.66; 95% CI, 0.51-0.86). The preventive attributes of reduced alcohol intake were consistently observed across various subgroups of participants.

**Conclusions and Relevance:**

Results of this cohort study suggest that reducing alcohol consumption is associated with a decreased risk of future CVD, with the most pronounced benefits expected for angina and ischemic stroke.

## Key Points

**Question:**

Is a reduction in alcohol consumption among people with previously heavy drinking associated with a decrease in major adverse cardiovascular events?

**Findings:**

In this cohort study of 21 011 individuals with heavy alcohol consumption at baseline, lowering alcohol intake to mild to moderate level was associated with a 23% reduction in the risk of major adverse cardiovascular events compared with sustained heavy drinking. The most substantial risk reduction was observed in the outcomes of angina and ischemic stroke.

**Meaning:**

Findings of this study provide crucial evidence of the cardiovascular benefits of reducing alcohol consumption in people who drink heavily.

## Teaser

This cohort study examines the association of reduced alcohol consumption with risk of major cardiovascular events among individuals who drink heavily.

## Introduction

Alcohol consumption is one of the most prevalent behavioral factors in personal and public health. Previous cohort studies and meta-analyses have reported the potential association between alcohol consumption and future cardiovascular disease (CVD); however, the dose-response relationship has not been uniformly addressed.^1,2,3,4,5,6,7^ It is widely accepted that mild to moderate alcohol consumption provides modest protection against future CVD.^8^ However, this protective attribute of mild to moderate alcohol consumption varies by individual subtypes of CVD.^1,2,3,4,9^ For example, the incidence of coronary artery disease (CAD) consistently decreased over a range of alcohol consumption (starting from >2.5 g/d); otherwise, U- or J-shaped dose-response patterns were observed for CVD mortality and stroke incidence.^1^

Most previous studies of the association between alcohol consumption and future CVD used a single time point to estimate mean alcohol intake and defined nondrinkers as the control group.^1,2,3,4,5,7^ However, an individual’s drinking habits do not always remain the same over time.^10,11,12,13^ Therefore, the association of alcohol consumption on future CVD should be investigated in the context of habitual change rather than fixed behavior. This methodological approach could provide practical evidence for clinicians who often encounter questions regarding the cardiovascular benefits of changing drinking behaviors. Notably, there is a lack of studies demonstrating the cardiovascular benefits of reduced alcohol consumption in people who drink heavily.^10,12^ Moreover, it is crucial to substantiate the result of behavioral changes in this population according to different CVD subtypes. Herein, we hypothesized that habitual changes in alcohol consumption among individuals with sustained heavy drinking would have a long-term role in future CVD outcomes. We conducted a nationwide population-based cohort study to investigate the association between reduced alcohol consumption and risk of major adverse cardiovascular events (MACEs) in people who drink heavily across different CVD subtypes.

## Methods

### Study Data Source

This nationwide population-based cohort study used data from the Korean National Health Insurance Service–Health Screening (NHIS-HEALS) database. The institutional review board of Chungbuk National University Hospital approved this study and waived the informed consent requirement because anonymized data were used. We complied with the Declaration of Helsinki^14^ and followed the Strengthening the Reporting of Observational Studies in Epidemiology (STROBE) reporting guideline.

The overall population cohort consisted of a random sample representing 10% of Korean residents with national health insurance coverage aged 40 to 79 years on December 31, 2002, who were also included in the 2002 to 2003 National Health Screening Program (NHSP). Given that the NHSP is a mandatory program that encompasses approximately 97.1% of adults, this cohort was representative of the entire adult population in South Korea. The variables contained in the NHIS-HEALS database included demographic characteristics, disease codes, prescription records, income status, and mortality information. Personal records of blood pressure readings, anthropometric measurements, medical and family histories, lifestyle behaviors, and laboratory results were systematically collected using the NHSP. Lifestyle behavioral factors, including alcohol consumption, were assessed by trained investigators using self-reported questionnaires. Details of the study data source have been published previously.^15^ The complete dataset, approved by the NHIS, was analyzed between February and May 2023.

### Study Population

**Figure 1.**
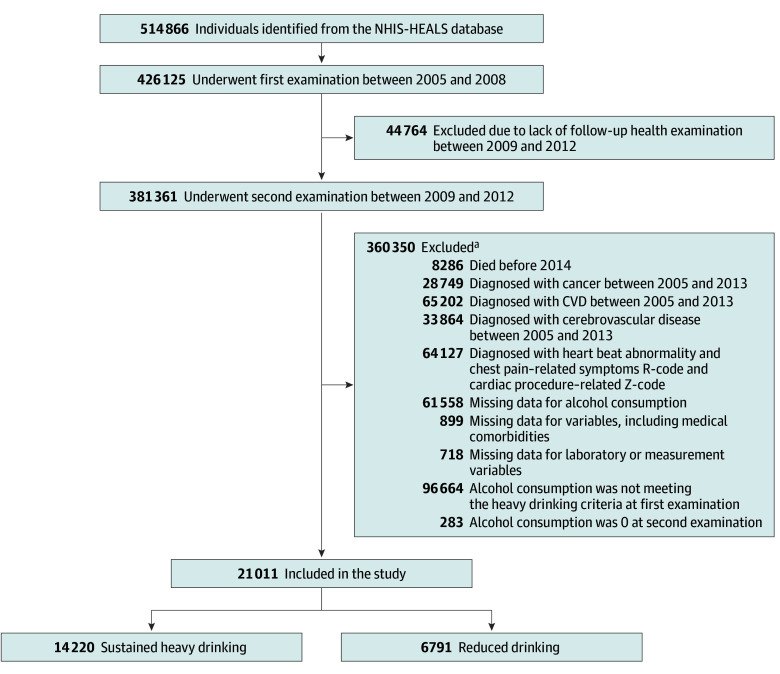
Flowchart of the Study Population CVD indicates cardiovascular disease; NHIS-HEALS, National Health Insurance Service–Health Screening. ^a^Individuals could be excluded for multiple reasons.

Figure 1 shows the flowchart of participant enrollment. Individuals who underwent the first medical checkup between 2005 and 2008 were included at baseline. The prespecified exclusion criteria are shown in Figure 1. Participants who abstained from alcohol drinking during the second checkup period were excluded, as the presence of the sick-quitter effect could confound the outcomes of alcohol-related behavioral change.^13^ Self-reported questionnaires were used to assess alcohol consumption amounts in participants who underwent health examinations over 2 consecutive periods (first period: 2005-2008; second period: 2009-2012).

eTable 1 in Supplement 1 shows the alcohol consumption calculation process. The risk level of alcohol consumption was defined using the mean intake amount during each health examination period according to the National Institute on Alcohol Abuse and Alcoholism (NIAAA) criteria, as previously described.^16,17^ Based on the NIAAA criteria, heavy drinking was defined as more than 4 drinks (56 g) per day or more than 14 drinks (196 g) per week for males and more than 3 drinks (42 g) per day or more than 7 drinks (98 g) per week for females (eTable 2 in Supplement 1). All study participants exhibited heavy drinking during the first health examination period (2005-2008) and were further categorized into 2 groups based on their alcohol consumption levels during the second health examination period (2009-2012): sustained heavy drinking and reduced drinking.

### Variables

Baseline demographic characteristics and laboratory findings were incorporated into the analysis as confounding variables. Specifically, the following variables were included as potential confounders: age; sex; body mass index (BMI; calculated as weight in kilograms divided by height in meters squared); obesity; systolic blood pressure; smoking status; level of physical activity; medical comorbidities of hypertension, diabetes, dyslipidemia, heart failure, chronic kidney disease (CKD), and atrial fibrillation (AF); laboratory results, including total cholesterol, low-density lipoprotein cholesterol (LDL-C), hemoglobin, glucose, serum creatinine, and alanine aminotransferase; modified Charlson Comorbidity Index (CCI; score range: 0-9, with higher scores indicating greater complexity of medical comorbidity); and income status. Medical comorbidities were primarily defined by *International Statistical Classification of Diseases and Related Health Problems, Tenth Revision (ICD-10)* diagnostic codes, supplemented by questionnaire-based responses in both health examination periods and a subsequent washout period. Smoking status and physical activity level were collected from the self-reported questionnaires. The definition of cardiovascular risk factors incorporated the potential changes observed during the baseline health examination periods to the subsequent washout period. Laboratory data were collected from the date closest to the index date of the second health examination period. Missing LDL-C values were imputed using the Friedewald equation. Details on the confounding variables are provided in eTable 2 in Supplement 1.

### Study Outcomes 

The primary outcome was the occurrence of MACEs until December 31, 2019. Defined as a composite outcome, MACEs included nonfatal myocardial infarction (MI) or angina requiring revascularization, any stroke accompanied by hospitalization, and all-cause death with corresponding *ICD-10* codes and death records. Procedure codes for coronary revascularization, including both interventional and surgical therapies, were incorporated to enhance the diagnostic accuracy of clinically relevant CAD.^18,19^

Secondary outcomes consisted of each component of the primary MACE composite. The index date of follow-up was the last screening date in the second health examination period. The study end date was the date that 1 of the following events occurred: (1) development of a cardiovascular outcome episode, (2) death, or (3) latest hospital visit of participants who remained free of outcome events. The first year following the end of the second health examination period up to December 31, 2013, was established as the washout period to minimize the potential lag of heavy alcohol consumption (mean [SD] washout duration, 23.0 [9.9] months).

### Statistical Analysis

Summary statistics were expressed as mean (SD) or number (%). The differences among groups were evaluated using an unpaired, 2-tailed *t* test for continuous variables and χ^2^ test for categorical variables. To minimize the implications of potential confounders, propensity score matching (PSM) analysis was performed using a logistic regression model with the nearest-neighbor method. The propensity score was calculated including potential confounders within the baseline covariates (eMethods in Supplement 1). The maximum absolute standardized difference was calculated, and values less than 0.1 indicated a negligible difference. The crude incidence rate was calculated as the number of events per 100 000 person-years. Cumulative incidence curves for MACEs were generated using the Kaplan-Meier method with the log-rank test. Hazard ratios (HRs) and 95% CIs of the study end points were estimated using multivariate Cox proportional hazards regression models. Subgroup analyses were conducted for MACEs using the following variables: age, sex, BMI, physical activity level, income status, medical comorbidities, modified CCI, and washout duration. Sensitivity analysis was performed using an alternative PSM and excluding measurement variables potentially modifiable by changes in alcohol consumption.

All statistical analyses were performed using RStudio, version 4.3.1 (R Project for Statistical Computing). All *P* values were 2-tailed, and *P* < .05 indicated statistical significance.

## Results

### Baseline Characteristics and Laboratory Findings

Among the 21 011 included participants with heavy alcohol consumption at baseline (18 963 males [90.3%], 2048 females [9.7%]; mean [SD] age, 56.08 [6.16] years), 14 220 (67.7%) sustained heavy drinking, whereas 6791 (32.2%) reduced drinking to a mild to moderate level (Table 1 and Figure 1). Table 1 shows the baseline characteristics of the study population. Before PSM, the sustained heavy drinking group showed lower mean (SD) age (55.76 [6.01] vs 56.74 [6.41] years; *P* < .001) and had a greater proportion of males (93.7% vs 83.1%; *P* < .001) compared with the reduced drinking group.

**Table 1.** Baseline Participant Characteristics by Group Before and After Propensity Score Matching (PSM)

Characteristic	Overall population (N = 21 011)				PSM population (n = 13 582)			
	Sustained heavy drinking group, No. (%) (n = 14 220)	Reduced drinking group, No. (%) (n = 6791)	*P* value	ASD	Sustained heavy drinking group, No. (%) (n = 6791)	Reduced drinking group, No. (%) (n = 6791)	*P* value	ASD
Age, mean (SD), y	55.76 (6.01)	56.74 (6.41)	<.001	0.158	57.00 (6.85)	56.74 (6.41)	.02	0.039
Sex								
Male	13 320 (93.7)	5643 (83.1)	<.001	0.335	5897 (86.8)	5643 (83.1)	<.001	0.105
Female	900 (6.3)	1148 (16.9)			894 (13.2)	1148 (16.9)		
BMI, mean (SD)	24.57 (2.78)	24.28 (2.77)	<.001	0.104	24.29 (2.76)	24.28 (2.77)	.94	<0.001
Obesity	6047 (42.5)	2620 (38.6)	<.001	0.088	2612 (38.5)	2620 (38.6)	.95	0.006
SBP, mean (SD), mm Hg	127.45 (14.20)	125.85 (14.39)	<.001	0.112	126.20 (14.09)	125.85 (14.39)	.16	0.024
Laboratory results								
TC, mean (SD), mg/dL	200.06 (35.86)	200.16 (35.66)	.84	0.003	199.64 (35.90)	200.16 (35.66)	.40	0.015
LDL-C, mean (SD), mg/dL	113.30 (35.56)	116.21 (35.81)	<.001	0.082	115.65 (36.41)	116.21 (35.81)	.37	0.015
Hemoglobin, mean (SD), g/dL	14.84 (1.23)	14.58 (1.32)	<.001	0.202	14.64 (1.31)	14.58 (1.32)	.01	0.043
Glucose, mean (SD), mg/dL	105.65 (26.46)	103.59 (25.17)	<.001	0.080	104.06 (24.94)	103.59 (25.17)	.28	0.019
Serum creatinine, mean (SD), mg/dL	1.00 (0.66)	0.99 (0.67)	.06	0.027	1.00 (0.71)	0.99 (0.67)	.29	0.018
ALT, mean (SD), IU/L	28.93 (21.22)	26.75 (19.93)	<.001	0.106	26.95 (19.61)	26.75 (19.93)	.57	0.010
Smoking status								
Nonsmoker	3547 (24.9)	2551 (37.6)	<.001	0.279	2436 (35.9)	2551 (37.6)	.10	0.037
Past	4975 (35.0)	2092 (30.8)			2120 (31.2)	2092 (30.8)		
Current	5698 (40.1)	2148 (31.6)			2235 (32.9)	2148 (31.6)		
Physical activity level^a^								
Less active	6638 (46.7)	3064 (45.1)	.10	0.031	3016 (44.4)	3064 (45.1)	.71	0.014
Active	4137 (29.1)	2037 (30.0)			2068 (30.5)	2037 (30.0)		
Highly active	3445 (24.2)	1690 (24.9)			1707 (25.1)	1690 (24.9)		
Medical comorbidity								
Hypertension	5468 (38.5)	2561 (37.7)	.31	0.015	2609 (38.4)	2561 (37.7)	.41	0.015
Diabetes	3508 (24.7)	1592 (23.4)	.055	0.029	1671 (24.6)	1592 (23.4)	.12	0.027
Dyslipidemia	5961 (41.9)	2815 (41.5)	.53	0.009	2793 (41.1)	2815 (41.5)	.71	0.007
Heart failure	113 (0.8)	53 (0.8)	.98	0.002	50 (0.7)	53 (0.8)	.84	0.005
CKD	867 (6.1)	490 (7.2)	.002	0.045	502 (7.4)	490 (7.2)	.72	0.007
AF	102 (0.7)	51 (0.8)	.86	0.004	54 (0.8)	51 (0.8)	.85	0.005
Modified CCI								
0	3366 (23.7)	1585 (23.3)	.75	0.030	1594 (23.5)	1585 (23.3)	.97	0.023
1	4027(28.3)	1894 (27.9)			1882 (27.7)	1894 (27.9)		
2	3238 (22.8)	1612 (23.7)			1578 (23.2)	1612 (23.7)		
3	2199 (15.5)	1009 (14.9)			1050 (15.5)	1009 (14.9)		
4	1008 (7.1)	499 (7.3)			500 (7.4)	499 (7.3)		
5	307 (2.2)	154 (2.3)			152 (2.2)	154 (2.3)		
6	58 (0.4)	29 (0.4)			29 (0.4)	29 (0.4)		
≥7	17 (0.1)	9 (0.1)			6 (0.1)	9 (0.1)		
Income status^b^								
Medicaid beneficiaries	11 (0.1)	11 (0.2)	<.001	0.121	10 (0.1)	11 (0.2)	.62	0.023
Low	2087 (14.7)	1220 (18.0)			1167 (17.2)	1220 (18.0)		
Middle	4102 (28.8)	2106 (31.0)			2155 (31.7)	2106 (31.0)		
High	8020 (56.4)	3454 (50.9)			3459 (50.9)	3454 (50.9)		

Abbreviations: AF, atrial fibrillation; ALT, alanine aminotransferase; ASD, absolute standardized difference; BMI, body mass index (calculated as weight in kilograms divided by height in meters squared); CCI, Charlson Comorbidity Index (score range: 0-9, with higher scores indicating greater complexity of medical comorbidity); CKD, chronic kidney disease; LDL-C, low-density lipoprotein cholesterol; MET, metabolic equivalent task; SBP, systolic blood pressure; TC, total cholesterol.

SI conversion factors: To convert ALT to microkatals per liter, multiply by 0.0167; serum creatinine to micromoles per liter, multiply by 88.4; glucose to millimoles per liter, multiply by 0.0555; hemoglobin level to gram per liter, multiply by 10.0; and LDL-C and TC to millimoles per liter, multiply by 0.0259.

^a^ Physical activity: less active indicates 0 to 500 MET-min/wk; active, 501 to 1000 MET-min/wk; and highly active, more than 1000 MET-min/wk.

^b^ Income status: low indicates 30th percentile or less; middle, more than 30th to less than 70th percentile; and high, 70th percentile or greater.

Most clinical variables, including BMI; systolic blood pressure; and hemoglobin, glucose, and alanine aminotransferase levels, were higher in the sustained heavy drinking group. The sustained heavy drinking group consisted of a greater proportion of individuals with current smoking, obesity, and high-income status. Conversely, the reduced drinking group had a higher incidence of CKD (7.2% vs 6.1%; *P* = .002) and elevated mean (SD) LDL-C levels (116.21 [35.81] vs 113.30 [35.56] mg/dL; *P* < .001; to convert to millimoles per liter, multiply by 0.0259) than the sustained heavy drinking group. After PSM, all variables were well balanced between the groups except for age, sex, and hemoglobin level. The maximum absolute standardized difference remained below 0.1 for all variables except for sex.

### Main Outcomes

**Figure 2.**
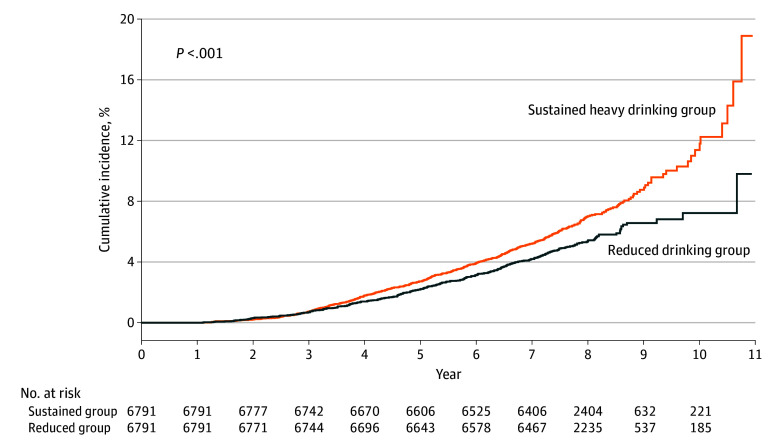
Cumulative Incidence Curves of Sustained Heavy Drinking and Reduced Drinking Groups After Propensity Score Matching

Over the follow-up duration of 162 378 person-years, 899 participants (6.3%) in the sustained heavy drinking group and 354 (5.2%) in the reduced drinking group developed MACEs. Figure 2 shows the Kaplan-Meier survival curve of the cumulative MACE incidence according to changes in alcohol consumption after PSM. The sustained heavy drinking group showed a significantly higher incidence of MACEs than the reduced drinking group (log-rank *P* < .001). The Kaplan-Meier curve of cumulative MACE incidence started to diverge after 3 years, and the difference gradually increased over time. The incidence rate of MACEs was 1.21 times higher in the sustained heavy drinking group than in the reduced drinking group (817 vs 675 per 100 000 person-years; log-rank *P* = .003) (Table 2). The reduced drinking group exhibited a 23% lower risk of MACEs (crude HR, 0.83 [95% CI, 0.74-0.94]; PSM HR, 0.77 [95% CI, 0.67-0.88]) than the sustained heavy drinking group.

**Table 2.** Hazard Ratios (HRs) for Primary and Secondary Outcomes by Group

Groups	No. of participants	No. of events	Person-years	Incidence rate per 100 000 person-years	Crude HR (95% CI)^a^	PSM HR (95% CI)^a^^,^^b^
**MACEs**						
Sustained heavy drinking	14 220	899	109 996	817	1 [Reference]	1 [Reference]
Reduced drinking	6791	354	52 382	675	0.83 (0.74-0.94)	0.77 (0.67-0.88)
**CAD**						
Sustained heavy drinking	14 220	196	110 821	177	1 [Reference]	1 [Reference]
Reduced drinking	6791	65	52 691	123	0.70 (0.53-0.92)	0.71 (0.52-0.98)
**Nonfatal MI**						
Sustained heavy drinking	14 220	76	111 175	68	1 [Reference]	1 [Reference]
Reduced drinking	6791	22	52 836	42	0.61 (0.38-0.98)	0.70 (0.41-1.20)
**Angina**						
Sustained heavy drinking	14 220	190	110 838	171	1 [Reference]	1 [Reference]
Reduced drinking	6791	62	52 699	118	0.69 (0.52-0.92)	0.70 (0.51-0.97)
**Any stroke**						
Sustained heavy drinking	14 220	344	110 563	311	1 [Reference]	1 [Reference]
Reduced drinking	6791	122	52 581	232	0.75 (0.61-0.92)	0.72 (0.57-0.91)
**Hemorrhagic stroke**						
Sustained heavy drinking	14 220	75	111 259	67	1 [Reference]	1 [Reference]
Reduced drinking	6791	32	52 819	61	0.91 (0.60-1.38)	0.91 (0.56-1.46)
**Ischemic stroke**						
Sustained heavy drinking	14 220	287	110 683	259	1 [Reference]	1 [Reference]
Reduced drinking	6791	94	52 643	179	0.69 (0.55-0.87)	0.66 (0.51-0.86)
**All-cause death**						
Sustained heavy drinking	14 220	427	111 412	383	1 [Reference]	1 [Reference]
Reduced drinking	6791	189	52 382	357	0.95 (0.80-1.12)	0.79 (0.66-0.96)

Abbreviations: CAD, coronary artery disease; MACEs, major adverse cardiovascular event; MI, myocardial infarction; PSM, propensity score matching.

^a^ The HRs were calculated using multivariate Cox proportional hazards regression models before and after PSM.

^b^ Adjusted for age, sex, body mass index, obesity, systolic blood pressure, total cholesterol, low-density lipoprotein cholesterol, hemoglobin, glucose, serum creatinine, alanine aminotransferase, smoking status, physical activity, hypertension, diabetes, dyslipidemia, heart failure, chronic kidney disease, atrial fibrillation, modified Charlson Comorbidity Index, and income status.

In the secondary outcome analyses (Table 2), the cardiovascular benefits of reduced alcohol consumption varied according to specific CVD subtypes. The risks of CAD (PSM HR, 0.71; 95% CI, 0.52-0.98), angina (PSM HR, 0.70; 95% CI, 0.51-0.97), any stroke (PSM HR, 0.72; 95% CI, 0.57-0.91), ischemic stroke (PSM HR, 0.66; 95% CI, 0.51-0.86), and all-cause death (PSM HR, 0.79; 95% CI, 0.66-0.96) decreased significantly. However, these preventive properties were not observed in nonfatal MI or hemorrhagic stroke.

### Subgroup Analysis

**Figure 3.**
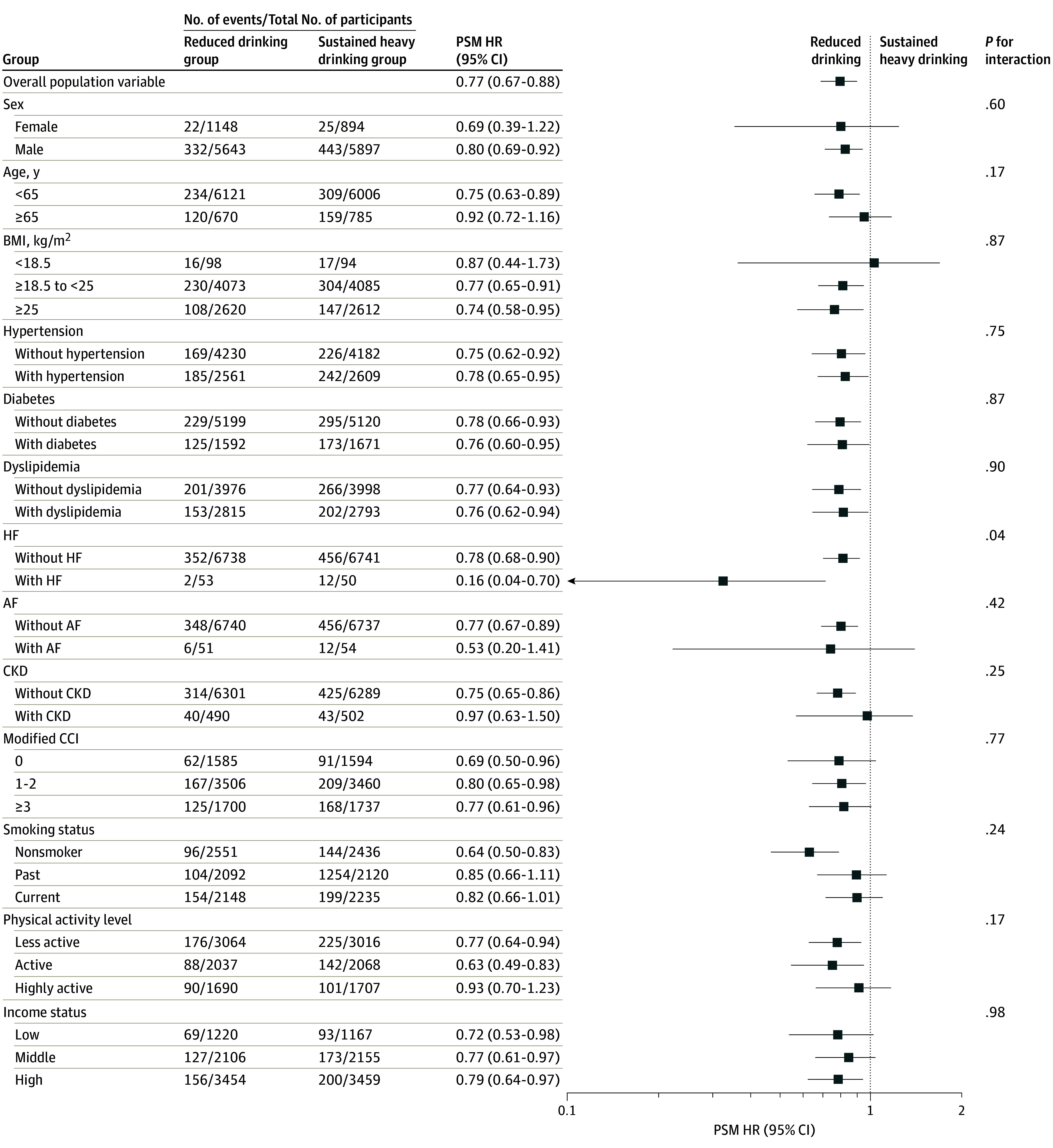
Subgroup Analysis of the Risk of Major Adverse Cardiovascular Events Between Reduced Drinking vs Sustained Heavy Drinking Groups Hazard ratios (HRs) with respective 95% CIs are displayed on a logarithmic (log_2_) scale. AF indicates atrial fibrillation; BMI, body mass index (calculated as weight in kilograms divided by height in meters squared); CCI, Charlson Comorbidity Index; CKD, chronic kidney disease; HF, heart failure; and PSM, propensity score matching.

Figure 3 shows the results of the subgroup analysis. While nonsignificant interactions were observed except for prior heart failure (HF) (*P* for interaction = .04), the prevention of MACEs associated with transitioning to reduced drinking was prominent in the subgroups of males (PSM HR, 0.80; 95% CI, 0.69-0.92), younger than 65 years (PSM HR, 0.75; 95% CI, 0.63-0.89), BMI of 18.5 to less than 25 (PSM HR, 0.77; 95% CI, 0.65-0.91), BMI of 25 or higher (PSM HR, 0.74; 95% CI, 0.58-0.95), nonsmoking status (PSM HR, 0.64; 95% CI, 0.50-0.83), and less active (PSM HR, 0.77; 95% CI, 0.64-0.94) and active (PSM HR, 0.63; 95% CI, 0.49-0.83) physical levels. This direct association persisted among participants without specific comorbidities, such as AF (PSM HR, 0.77; 95% CI, 0.67-0.89) and CKD (PSM HR, 0.75; 95% CI, 0.65-0.86). Notably, the cardiovascular benefits of alcohol reduction were consistently observed regardless of modified CCI; income status; and certain medical conditions, including hypertension, diabetes, dyslipidemia, and HF. Additionally, no definite interaction was observed concerning the washout duration (eTable 3 in Supplement 1).

### Sensitivity Analysis With Alternative PSM

Additional PSM analysis was conducted by excluding variables potentially modifiable by changes in alcohol consumption (eTable 4 and eResults in Supplement 1). These findings did not alter the observed cardiovascular benefits associated with reduced alcohol consumption.

## Discussion

In this nationwide population-based cohort study, reduced alcohol consumption among people who drink heavily was associated with a lower risk of future cardiovascular events. Notably, habitual changes in heavy alcohol consumption from 2009 to 2012 were beneficial over the subsequent follow-up period of up to 10 years. Moreover, these benefits became increasingly evident approximately 3 years after the initiation of alcohol-related behavioral change. These preventive attributes were consistently observed across the various subgroups of people who drank heavily. The cardiovascular benefits of reduced alcohol intake presented differentially according to subtypes of CVD. More pronounced benefits were found, such as lower risk of ischemic stroke and ischemia-driven revascularization for angina. Collectively, these findings suggest that reducing alcohol consumption among people who drink heavily is potentially associated with a broad spectrum of benefits in future cardiovascular events and specific CVD subtypes.

### Biological Mechanism of Protection From Reduced Alcohol Intake

Many previous studies have reported a potential association between mild to moderate alcohol consumption and reduced cardiovascular risk; however, a causal association remains unclear. Several plausible biological mechanisms have been proposed to explain this phenomenon. Mild to moderate alcohol consumption orchestrates various biological processes that mitigate cardiovascular risk, including beneficial regulation of lipid profiles and fibrinolysis,^20^ decreased platelet aggregation,^21^ improved endothelial dysfunction,^22^ reduced vascular inflammation,^23^ and insulin resistance.^24^ Furthermore, mild to moderate alcohol consumption can play a role in lower stress-associated neurobiological activity,^25^ a neural underpinning of atherosclerotic CVD.^26,27^ These biological mechanisms of mild to moderate alcohol consumption may collaborate synergistically to reduce the risk of future CVD. In contrast, heavy alcohol consumption is associated with increased future cardiovascular risk by promoting predisposing medical comorbidities, including hypertension, obesity, and sleep apnea. Based on this evidence, it can be hypothesized that reducing alcohol intake among people who drink heavily may be a factor in lower future cardiovascular risks. While this hypothesis remains to be thoroughly examined, conducting a large-scale prospective randomized clinical trial is nearly impossible because of ethical concerns regarding the establishment of individuals with sustained heavy drinking as the control group. Given these constraints, this cohort study provides crucial evidence regarding this high-risk population.

### Differential Implications for Coronary Vascular and Cerebrovascular Beds

Although mild to moderate alcohol consumption is generally considered to be protective against CAD,^1,3,4,8,9,28^ it remains unclear whether comparable benefits are expected from habitual changes in people who drink heavily. The present study found that reducing alcohol consumption in people who drink heavily is associated with lower risk of future CAD. This study introduced a novel perspective by conceptualizing alcohol consumption as a dynamic behavior and enables the exploration of the clinical implications of alcohol-related behavioral changes. The key findings align with contemporary guidelines, supporting the potential benefit of mild to moderate alcohol consumption in CAD.^29,30^ Reduced alcohol consumption in individuals who drink heavily was associated with a 29% decrease in the overall risk of CAD (PSM HR, 0.71; 95% CI, 0.52-0.98), which was mostly accounted for by a decrease in stable or unstable angina. The benefit of mild to moderate alcohol consumption in participants with CAD appeared to be less prominent in MI occurrence. This discrepancy between angina and MI has been documented in previous studies.^2,4^ Although the detailed pathophysiological mechanisms remain to be clarified, the present study highlighted the divergent outcomes of reduced alcohol consumption across distinct subtypes of CAD.

The potential neuroprotection of mild to moderate alcohol consumption has been reported based on a U-shaped or curvilinear dose-response relationship for the incidence or mortality of stroke.^1,3,4,9^ However, no studies have addressed the implications of alcohol-related behavioral changes in heavy drinking across different stroke subtypes. In the present study, we conducted a dedicated analysis to explore the differential benefits of less alcohol intake by individual stroke subtypes. Reduced alcohol consumption in individuals who drank heavily resulted in a 34% decrease in risk of ischemic stroke (PSM HR, 0.66; 95% CI, 0.51-0.86); however, this preventive association did not correspond to hemorrhagic stroke. Theoretically, this discrepancy may be attributed to the altered platelet function and coagulation factors following moderate alcohol consumption that leads to higher susceptibility to hemorrhagic stroke.^31^ The findings from the present study are congruous with those of previous studies that reported on the differential association between alcohol consumption and distinct stroke types.^32,33^ Additionally, a previous study demonstrated that reduced drinking was associated with lower risk of AF.^34^ Taken together, these findings suggest a potential to reduce ischemic events but a neutral or inverse association with hemorrhagic events. Another important aspect to consider is that ischemic stroke is a multifactorial and heterogeneous disease. Given its intricate pathophysiological processes, a substantial knowledge gap persists concerning the divergent implications of mild to moderate alcohol consumption according to different subtypes of ischemic stroke. Future studies should clarify these issues to enhance the understanding of the cerebrovascular implications of alcohol-reduction interventions.

### Strengths and Limitations

This study has some strengths. To our knowledge, this study was the first to demonstrate the clinical implications of habitual changes in alcohol consumption among people who drink heavily for the future risk of cardiovascular events. Reduced alcohol consumption in individuals with sustained heavy drinking was associated with lower incidence of angina and ischemic stroke. These findings provide practical insights into the development of diagnostic and therapeutic strategies to effectively manage heavy drinking in routine clinical practice. Clinicians may be apprehensive about the substantially higher risk of angina or ischemic stroke in people who drink heavily. Furthermore, clinicians can offer personalized guidance to reduce alcohol consumption and effectively manage specific subtypes of alcohol-attributable CVDs.

This study has certain limitations. First, assessment that uses self-reported alcohol intake has the potential to result in misclassification. Second, we were unable to detect additional alcohol-related behavioral changes before the first health examination period and beyond the second health examination period. We assumed that the alcohol consumption captured during the second health examination period remained unchanged during the follow-up. Third, the study population was composed exclusively of South Koreans. Future studies should include diverse ethnicities to establish global evidence and ascertain the generalizability of the findings. Fourth, the study design did not allow for the identification of a causal association. While a randomized clinical trial is precluded by ethical concerns, the time-exposure study design provided the opportunity to explore potential biological associations. Fifth, we were unable to assess individual variations in alcohol metabolism, such as single-nucleotide variants (formerly single-nucleotide polymorphisms). Sixth, detailed information on the cause of death and broader socioeconomic status was unavailable in the NHIS-HEALS database. Seventh, despite conducting a PSM analysis, it remains possible that residual confounding factors might have been included in the analysis. Eighth, the study design might not inherently exclude survivor bias. However, examining 2 distinct health examination periods is crucial to investigate changes in lifestyle behavior, such as alcohol consumption, and results should be interpreted with consideration of this aspect. Nine, absolute abstinence from heavy alcohol consumption was not thoroughly evaluated due to the limited sample size and event number of abstainers.

## Conclusions

This nationwide population-based cohort study provided crucial evidence of the cardiovascular benefits of reduced alcohol consumption, with the most substantial risk reduction observed in stable or unstable angina and ischemic stroke. The findings highlight the importance of disease-specific personalized guidance and targeted public health initiatives to promote alcohol intake–reduction strategies. These comprehensive interventions play a pivotal role in mitigating the risk of alcohol-attributable CVDs among people who drink heavily.

## Notes

Supplement 1. **eMethods.** Potential Confounding Variables Included in the PSM Analysis **eResults.** Sensitivity Analysis With Alternative PSM **eTable 1.** Overall Process of Alcohol Consumption Calculation **eTable 2.** Operational Definitions of Alcohol Consumption Levels, Confounding Variables and Primary Outcome **eTable 3.** Subgroup Analysis of MACEs Risk Between Reduced Drinking and Sustained Heavy Drinking Stratified by Washout Duration **eTable 4.** Adjusted Hazards Ratios for Primary and Secondary Outcomes in Alternative PSM Analysis Excluding Modifiable Measurement Variables
eReferences

Supplement 2.
Data Sharing Statement
