# Supplementary material for: Reduced Alcohol Consumption and Major Adverse Cardiovascular Events Among Individuals With Previously High Alcohol Consumption
Source: JAMA Netw Open. 2024 Mar 28;7(3):e244013. doi: 10.1001/jamanetworkopen.2024.4013 (PMC10979316; doi:10.1001/jamanetworkopen.2024.4013)
Supplement: Supplement 1. — eMethods. Potential Confounding Variables Included in the PSM Analysis eResults. Sensitivity Analysis With Alternative PSM eTable 1. Overall Process of Alcohol Consumption Calculation eTable 2. Operational Definitions of Alcohol Consumption Levels, Confounding Variables and Primary Outcome eTable 3. Subgroup Analysis of MACEs Risk Between Reduced Drinking and Sustained Heavy Drinking Stratified by Washout Duration eTable 4. Adjusted Hazards Ratios for Primary and Secondary Outcomes in Alternative PSM Analysis Excluding Modifiable Measurement Variables eReferences [file jamanetwopen-e244013-s001.pdf]

Supplemental Online Content

Kang DO, Lee DI, Roh SY, et al. Reduced alcohol consumption and major adverse cardiovascular events among individuals with previously high alcohol consumption. *JAMA Netw Open*. 2024;7(3):e244013. doi:10.1001/jamanetworkopen.2024.4013

eMethods. Potential Confounding Variables Included in the PSM Analysis

eResults. Sensitivity Analysis With Alternative PSM

eTable 1. Overall Process of Alcohol Consumption Calculation

eTable 2. Operational Definitions of Alcohol Consumption Levels, Confounding Variables and Primary Outcome

eTable 3. Subgroup Analysis of MACE Risk Between Reduced Drinking and Sustained Heavy Drinking Stratified by Washout Duration

eTable 4. Adjusted Hazards Ratios for Primary and Secondary Outcomes in Alternative PSM Analysis Excluding Modifiable Measurement Variables

eReferences

This supplemental material has been provided by the authors to give readers additional information about their work.

© 2024 Kang DO et al. *JAMA Network Open*

eMethods. Potential Confounding Variables Included in the PSM Analysis

To minimize the impact of potential confounders, propensity score-matching (PSM) analysis was performed using a logistic regression model with nearest neighbor method. The propensity score (PS) was calculated using the baseline covariates including age, sex, body mass index (BMI), obesity, systolic blood pressure (SBP), total cholesterol (TC), low-density lipoprotein cholesterol (LDL-C), hemoglobin, glucose, serum creatinine (sCr), alanine aminotransferase (ALT), smoking status, physical activity, hypertension, diabetes mellitus (DM), dyslipidemia, heart failure (HF), chronic kidney disease (CKD), atrial fibrillation (AF), modified Charlson Comorbidity Index (CCI), and income status.

© 2024 Kang DO et al. *JAMA Network Open*

eResults. Sensitivity Analysis With Alternative PSM

We conducted an additional PSM analysis by excluding potentially modifiable variables influenced by change in alcohol consumption - such as measurements of blood pressure, lipid levels, blood glucose indices, hemoglobin, and renal and liver functional parameters (Supplementary Table 4). In this sensitivity analysis, the reduced drinking group consistently showed a significantly lower risk of major adverse cardiovascular events (MACE) than the sustained drinking group (PSM hazard ratios [HRs] [95% confidence interval (CI)]: 0.732 (0.639–0.839)). Reduced alcohol intake demonstrated a significant preventive association in the secondary outcome analysis, particularly for coronary artery disease (CAD), angina, any stroke, ischemic stroke and all-cause death (PSM-HRs [95% CI]: 0.690 [0.503–0.946], 0.695 [0.503–0.960], 0.686 [0.545–0.863], 0.624 [0.482–0.807], and 0.760 [0.630–0.918], respectively). These findings suggest that excluding modifiable measurement variables from the PS calculation and matching did not significantly alter the observed cardiovascular benefits associated with reduced alcohol consumption.

© 2024 Kang DO et al. *JAMA Network Open*

eTable 1. Overall Process of Alcohol Consumption Calculation

Questionnaire of alcohol consumption habit in Korean National Insurance Service-Health Screening (English translated version)

Questions for alcohol consumption (2005–2008)

Q1) What is your drinking habit most likely?

① (Almost) never drink. ② Drink about 2-3 times a month. ③ Drink 1-2 times a week.

④ Drink 3-4 times a week. ⑤ Drink almost every day.

Q2) If you are drinking, how much do you consume in one occasion?

① Less than half a bottle of soju. ② One bottle of soju. ③ One and a half bottles of soju.

④ Two or more bottles of soju.

Questions for alcohol consumption (2009–2012)

Please read the questions below and fill in the information which corresponds to your current status.

Q1) On average, how many days a week do you drink?

□ 0 □ 1 □ 2 □ 3 □ 4 □ 5 □ 6 □ 7

Q2) How much do you usually drink a day when you drink (cup)? ________

(※ We count by each glass, regardless of the liquor type. One canned beer (355 cc) is equivalent to 1.6 cups of beer)

Note) Alcohol content was calculated by multiplying the drinking frequency by the number of standard drinks per serving. A standard drink in Korea, corresponding to one cup of each alcohol type, contains

© 2024 Kang DO et al. *JAMA Network Open*

seven grams of alcohol.[1-3] The Ministry of Health and Welfare of Republic of Korea has defined standard drink as seven grams of pure alcohol content for Soju and beer in 2020.[1] Moreover, the Korean Customs Service and Korean Alcohol Liquor Industry Association provided that Soju and beer were the most commonly consumed liquors in the Republic of Korea.[2]
**Reference** [1-3]<br>1. Action plan for the prevention of harmful effects of drinking in South Korea (http://ilovegb.kr/new2019/download/policy_2020_03.pdf)<br>2. Korean Customs Service and Korean Alcohol Liquor Industry Association (http://www.kalia.or.kr)<br>3. Lee SR, Choi EK, Jung JH, Han KD, Oh S, Lip GYH. Lower risk of stroke after alcohol abstinence in patients with incident atrial fibrillation: a nationwide population-based cohort study. Eur Heart J. 2021;42(46):4759-68.

© 2024 Kang DO et al. *JAMA Network Open*

eTable 2. Operational Definitions of Alcohol Consumption Levels, Confounding Variables and Primary Outcome

Category	ICD-10 codes and definitions	Assessment period
**Alcohol consumption levels**		
**Heavy**	Men: >4 drinks (56 g)/day or >14 drinks (196 g)/week	First and second health examinations
	Women: >3 drinks (42 g)/day or >7 (98 g) drinks/week	
**Moderate**	Men: ≤4 drinks (56 g)/day and ≤14 drinks (196 g)/week	
	Women: ≤3 drinks (42 g)/day and ≤7 drinks (98 g)/week	
**None**	None: 0 g	
**Confounding variables**		
**Smoking status**	Current smoker / past smoker / non-smoker	Index date
**Obesity**	Underweight: <18.5 kg/m^2	Index date
	Normal to overweight: ≥18.5 to <25.0 kg/m^2	
	Obesity: ≥25.0 kg/m^2	
**Physical activity**	Insufficiently active: 0–499 MET-minutes/week	Index date
	Active: 500–1000 MET-minutes/week	

© 2024 Kang DO et al. *JAMA Network Open*

	Highly active: ≥1,000 MET-minutes/week	
**Income status**	Low level: ≤30th percentile<br>Middle level: >30th to ≤70th percentile<br>High level: >70th percentile	Index date
**Hypertension**	I10-13, Admission or outpatient consultation ≥1; relevant medical history reported in the self-questionnaire	Between 2005 and 2013
**Diabetes mellitus**	E11-14, Admission or outpatient consultation ≥1; relevant medical history reported in the self-questionnaire	Between 2005 and 2013
**Dyslipidaemia**	E78, Admission or outpatient consultation ≥1; relevant medical history reported in the self-questionnaire	Between 2005 and 2013
**Heart failure**	I50, Admission or outpatient consultation ≥1; relevant medical history reported in the self-questionnaire	Between 2005 and 2013
**Atrial fibrillation**	I48, Admission or outpatient consultation ≥1; relevant medical history reported in the self-questionnaire	Between 2005 and 2013
**Chronic kidney disease**	eGFR <60 mL/min/1.73 m^2	Index date

© 2024 Kang DO et al. *JAMA Network Open*

	eGFR is calculated using the Chronic Kidney Disease–Epidemiology Collaboration equation as follows: eGFR = 141× min (Scr/κ, 1)$^{\alpha}$ × max (Scr/ κ, 1)$^{-1.209}$ × 0.993^{Age} × 1.018 [if female] × 1.159 [if black]	
**Modified CCI**	Excluding MI and cerebrovascular disease from the original scoring system[4]	Between 2005 and 2013
**Primary outcome**		
**Major adverse cardiovascular events**	Coronary artery disease; non-fatal MI (I21-I22) or angina (I200, I208-I209, I248-I1251, I255-I256, and I258-I259) undergoing revascularization therapy (M6551, M6552, M6561, M6562, M6563, M6564, M6571, M6572, M6633, M6634, O1641, O1642, O1647, OA641, OA642, and OA647)	
	Any stroke; ischemic stroke (I63) or hemorrhagic stroke (I60-62) accompanied by hospitalization	
	All-cause death	

© 2024 Kang DO et al. *JAMA Network Open*

Index date refers to the final examination date in the second period (years 2009-2012). Abbreviations: AF, atrial fibrillation; eGFR, estimated glomerular filtration rate; CAD, coronary artery disease; CCI, Charlson comorbidity index; MET, metabolic equivalent of task; MI, myocardial infarction; sCr, serum creatinine

© 2024 Kang DO et al. *JAMA Network Open*

eTable 3. Subgroup Analysis of MACE Risk Between Reduced Drinking and Sustained Heavy Drinking Stratified by Washout Duration

	**Reduced drinking (event / number)**	**Sustained heavy drinking (event / number)**	**PSM-HR* (95% CI)**	**$P_{interaction}$**
**Washout period**				0.498
Washout < 2 years	201 / 4,422	237 / 4,196	0.803 (0.665–0.969)	
Washout ≥ 2 years	153 / 2,369	231 / 2,595	0.729 (0.594–0.894)	

Adjusted hazard ratios are calculated using multivariate Cox proportional hazards regression models after PSM. *Adjusted for age, sex, BMI, obesity, SBP, TC, LDL-C, hemoglobin, glucose, creatinine, ALT, smoking status, physical activity, hypertension, DM, dyslipidemia, HF, CKD, AF, Modified CCI, and income status.

Abbreviations: AF, atrial fibrillation; ALT, alanine aminotransferase; BMI, body mass index; CCI, Charlson Comorbidity Index; CI, confidence interval; CKD, chronic kidney disease; DM, diabetes mellitus; HF, heart failure; HR, hazard ratio; LDL-C, low-density lipoprotein cholesterol; MACE, major adverse cardiovascular events; PSM, propensity score-matching; SBP, systolic blood pressure; TC, total cholesterol

© 2024 Kang DO et al. *JAMA Network Open*

eTable 4. Adjusted Hazards Ratios for Primary and Secondary Outcomes in Alternative PSM Analysis Excluding Modifiable Measurement Variables

		Number	Events	Person-years	Incidence rate (per 100,000 person-years)	PSM-HR* (95% CI)
**MACE**	Sustained	6,791	490	52,606	931	1 (Reference)
	Reduced	6,791	354	52,382	675	0.732 (0.639–0.839)
**CAD**	Sustained	6,791	95	53,022	179	1 (Reference)
	Reduced	6,791	65	52,691	123	0.690 (0.503–0.946)
Non-fatal MI	Sustained	6,791	35	53,221	66	1 (Reference)
	Reduced	6,791	22	52,836	42	0.641 (0.376–1.092)
Angina	Sustained	6,791	90	53,035	170	1 (Reference)
	Reduced	6,791	62	52,699	118	0.695 (0.503-0.960)

© 2024 Kang DO et al. *JAMA Network Open*

**Any stroke**	Sustained	6,791	180	52,890	340	1 (Reference)
	Reduced	6,791	122	52,581	232	0.686 (0.545–0.863)
Hemorrhagic stroke	Sustained	6,791	36	53,267	68	1 (Reference)
	Reduced	6,791	32	52,819	61	0.910 (0.565–1.466)
Ischemic stroke	Sustained	6,791	152	52,934	287	1 (Reference)
	Reduced	6,791	94	52,643	179	0.624 (0.482–0.807)
**All-cause death**	Sustained	6,791	255	53,321	478	1 (Reference)
	Reduced	6,791	189	52,382	357	0.760 (0.630–0.918)

Adjusted hazard ratios are calculated using multivariate Cox proportional hazards regression models after PSM. Incidence rates are displayed per 100,000 person-years. *Adjusted for age, sex, BMI, obesity, smoking status, physical activity, hypertension, DM, dyslipidemia, HF, CKD, AF, Modified CCI, and income status.

Abbreviations: AF, atrial fibrillation; BMI, body mass index; CCI, Charlson Comorbidity Index; CI, confidence interval; CKD, chronic kidney disease; DM, diabetes mellitus; HF, heart failure; HR, hazard ratio; MACE, major adverse cardiovascular events; MI, myocardial infarction

© 2024 Kang DO et al. *JAMA Network Open*

eReferences

1. Welfare MoHa. Action plan for the prevention of harmful effects of drinking in South Korea. Published 2019 Accessed. http://ilovegb.kr/new2019/download/policy_2020_03.pdf

2. Association KALI. Korean Alcohol Liquor Industry Association Accessed. http://www.kalia.or.kr

3. Lee SR, Choi EK, Jung JH, Han KD, Oh S, Lip GYH. Lower risk of stroke after alcohol abstinence in patients with incident atrial fibrillation: a nationwide population-based cohort study. *Eur Heart J.* 2021;42(46):4759-4768. doi:10.1093/eurheartj/ehab315

4. Charlson ME, Pompei P, Ales KL, MacKenzie CR. A new method of classifying prognostic comorbidity in longitudinal studies: development and validation. *J Chronic Dis.* 1987;40(5):373-383. doi:10.1016/0021-9681(87)90171-8
