# Supplementary material for: Reduced Alcohol Consumption and Major Adverse Cardiovascular Events Among Individuals With Previously High Alcohol Consumption
Source: JAMA Netw Open. 2024 Mar 28;7(3):e244013. doi: 10.1001/jamanetworkopen.2024.4013 (PMC10979316; doi:10.1001/jamanetworkopen.2024.4013)
Supplement: Supplement 2. — Data Sharing Statement [file jamanetwopen-e244013-s002.pdf]

Data Sharing Statement

Kang. Reduced Alcohol Consumption and Major Adverse Cardiovascular Events Among Individuals With Previously High Alcohol Consumption. *JAMA Netw Open*. Published March 28, 2024. doi:10.1001/jamanetworkopen.2024.4013

Data
Data available: Yes
Data types: Other (please specify)
Additional Information: The National Health Insurance Service of the Republic of Korea approved data sharing. The corresponding author has full access to the study data, and anonymized data will be available upon request from a qualified researcher.
How to access data: The National Health Insurance Service of the Republic of Korea approved data sharing. The corresponding author has full access to the study data, and request should be made to the corresponding author (dr.jinmanjung@gmail.com).
When available: With publication

Supporting Documents
Document types: None

Additional Information
Who can access the data: Anonymized data will be available upon request from a qualified researcher.
Types of analyses: Anonymized data will be available for post-hoc analysis purpose only.
Mechanisms of data availability: The data will be made available after approval of a proposal.
Any additional restrictions: N/A.
